# Clinical outcomes up to 9 years after [^18^F]flutemetamol amyloid-PET in a symptomatic memory clinic population

**DOI:** 10.1186/s13195-023-01351-1

**Published:** 2023-11-27

**Authors:** Lyduine E. Collij, Gill Farrar, Marissa Zwan, Elsmarieke van de Giessen, Rik Ossenkoppele, Frederik Barkhof, Annemieke J. M. Rozemuller, Yolande A. L. Pijnenburg, Wiesje M. van der Flier, Femke Bouwman

**Affiliations:** 1 Department of Radiology and Nuclear Medicine, Amsterdam UMC – location VUmc, Amsterdam, The Netherlands; 2 Amsterdam Neuroscience, Brain Imaging, Amsterdam, The Netherlands; 3 Clinical Memory Research Unit, Department of Clinical Sciences Malmö, Lund University, Lund, Sweden; 4 GE Healthcare, Amersham, UK; 5 Alzheimer Center and Department of Neurology, Amsterdam UMC – location VUmc, Amsterdam, The Netherlands; 6 Amsterdam Neuroscience, Neurodegeneration, Amsterdam, The Netherlands; 7 Centre for Medical Image Computing, and Queen Square Institute of Neurology, UCL, London, UK; 8 Department of Pathology, Amsterdam UMC – Location VUmc, Amsterdam, The Netherlands; 9 Department of Epidemiology and Data Science, Amsterdam UMC – location VUmc, Amsterdam, The Netherlands

**Keywords:** [^18^F]flutemetamol amyloid-PET, Early-onset dementia, Diagnosis, Survival, Neuropathology

## Abstract

**Background:**

Previous studies demonstrated increases in diagnostic confidence and change in patient management after amyloid-PET. However, studies investigating longitudinal outcomes over an extended period of time are limited. Therefore, we aimed to investigate clinical outcomes up to 9 years after amyloid-PET to support the clinical validity of the imaging technique.

**Methods:**

We analyzed longitudinal data from 200 patients (*M*_age_ = 61.8, 45.5% female, *M*_MMSE_ = 23.3) suspected of early-onset dementia that underwent [^18^F]flutemetamol-PET. Baseline amyloid status was determined through visual read (VR). Information on mortality was available with a mean follow-up of 6.7 years (range = 1.1–9.3). In a subset of 108 patients, longitudinal cognitive scores and clinical etiological diagnosis (eDx) at least 1 year after amyloid-PET acquisition were available (*M* = 3.06 years, range = 1.00–7.02). VR − and VR + patients were compared on mortality rates with Cox Hazard’s model, prevalence of stable eDx using chi-square test, and longitudinal cognition with linear mixed models. Neuropathological data was available for 4 patients (mean delay = 3.59 ± 1.82 years, range = 1.2–6.3).

**Results:**

At baseline, 184 (92.0%) patients were considered to have dementia. The majority of VR + patients had a primary etiological diagnosis of AD (122/128, 95.3%), while the VR − group consisted mostly of non-AD etiologies, most commonly frontotemporal lobar degeneration (30/72, 40.2%). Overall mortality rate was 48.5% and did not differ between VR − and VR + patients. eDx at follow-up was consistent with baseline diagnosis for 92/108 (85.2%) patients, with most changes observed in VR − cases (VR −  = 14/35, 40% vs VR +  = 2/73, 2.7%, *χ*^2^ = 26.03, *p* < 0.001), who at no time received an AD diagnosis. VR + patients declined faster than VR − patients based on MMSE (*β* =  − 1.17, *p* = 0.004), episodic memory (*β* =  − 0.78, *p* = 0.003), fluency (*β* =  − 1.44, *p* < 0.001), and attention scores (*β* = 16.76, *p* = 0.03). Amyloid-PET assessment was in line with *post-mortem* confirmation in all cases; two cases were VR + and showed widespread AD pathology, while the other two cases were VR − and showed limited amyloid pathology.

**Conclusion:**

In a symptomatic population, we observed that amyloid-status did not impact mortality rates, but is predictive of cognitive functioning over time across several domains. Also, we show particular validity for a negative amyloid-PET assessment, as these patients did not receive an AD diagnosis at follow-up.

## Introduction

Positron emission tomography (PET) imaging allows the in vivo visualization of the amyloid-β (Aβ) protein, a pathological hallmark of Alzheimer’s disease (AD) [1]. In a clinical setting, amyloid-PET images are visually assessed by trained readers, resulting in a binary classification of negative or positive for the presence of Aβ in the brain [2]. This straightforward approach has shown high clinical value, with previous studies demonstrating an increase in diagnostic confidence and change in patient diagnosis and management after amyloid-PET [3–5]. However, studies investigating longitudinal outcomes over an extended period of time to support the technique’s clinical validity are limited.

The strategic roadmap published in 2017 [6] and updated in 2021 [7] provides a methodological framework for the systematic validation of AD diagnostic biomarkers for the clinical routine. Importantly, it stated that clinical validity (phase 4) evidence or “real world performance” was incomplete for amyloid-PET imaging and reimbursement is lagging also due to the lack of this evidence. This phase of biomarker validation requires longitudinal studies in real-world patients, which assess clinically meaningful outcomes across three categories: (1) clinician-centered, (2) patient- and caregiver-centered, and (3) health economics-centered [8]. Most previous studies reported on evidence belonging to the first category, demonstrating a change in diagnosis in 19–79% of cases after amyloid-PET disclosure to the clinician [8]. However, while considered part of the primary aim of phase 4 studies, the long-term stability of clinical diagnosis or lack thereof has been scarcely reported. Regarding the second category, previous studies mainly reported distress or psychological impact after amyloid-PET status disclosure. Ramusino and colleagues (2021) highlight the lack of mortality rate studies in patients diagnosed with amyloid-PET(8), arguably the main patient-centered outcome. To date, only one study with adequate follow-up time has reported no increased risk of mortality in amyloid-positive dementia patients compared to their negative counterparts [9]. However, these results were based on a population-based observational study, which does not directly translate to a clinical setting.

In line with the appropriate use criteria (AUC) developed by the amyloid imaging task force (AIT) [10], we previously investigated the value of amyloid-PET in the work-up of patients suspected of early-onset dementia [5]. We reported a 19% change in diagnosis, an increase in diagnostic confidence, and 37% change in patient management after amyloid-PET disclosure [5]. Annual follow-up data ranging up to 9 years was collected for a large portion of this original population, resulting in a unique clinical data set to assess longitudinal outcomes.

To provide further evidence on the clinical validity of amyloid-PET, we assessed the longitudinal clinical and cognitive outcomes in the Dutch Flutemetamol Study [5] from the Alzheimer Center Amsterdam [11]. Here, we provide information on survival rates in our population stratified by amyloid status, stability in etiological diagnosis, rates of cognitive decline across several domains, and present *post-mortem* data where available.

## Methods

### Cohort

**Fig. 1:**
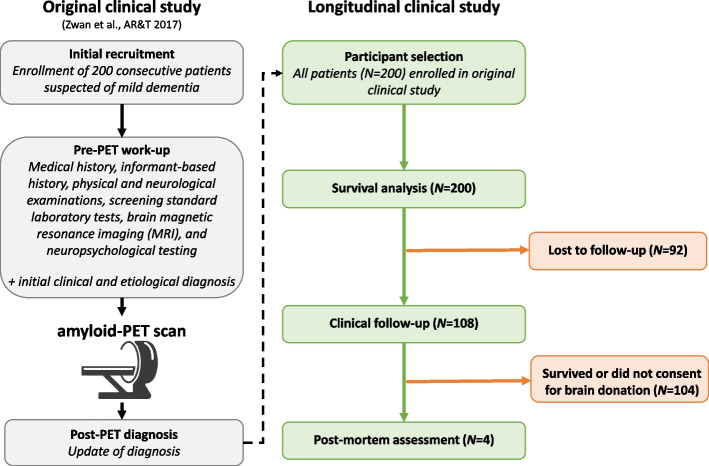
Flow diagram of study design and patient selection In the panel on the left (gray), the study design of the original clinical study is described [5]. In the panels on the right, the selection (green) and exclusion (orange) process of the current longitudinal study is shown

Longitudinal clinical data from 200 patients was collected from the Dutch Flutemetamol Study (DFS) [5] within the Amsterdam Dementia Cohort [11] from the Amsterdam University Medical Center. The cohort consists of patients who consecutively visited the VU University Medical Center Alzheimer Center in 2012–2014, at initial enrollment were suspected of mild dementia, had a Mini-Mental State Examination (MMSE) score of at least 18 to indicate competence for providing informed consent to participate in research, and underwent amyloid-PET as part of their diagnostic work-up. See Fig. 1 for an overview of the study design and patient selection.

This study was approved by the medical ethics review committee of the VU University Medical Center (reference number 2012/302).

### Participants

All patients received a standard dementia evaluation that included medical history, informant-based history, physical and neurological examinations, standard laboratory tests, brain magnetic resonance imaging (MRI), and neuropsychological testing. Clinical diagnosis was established by consensus in a multidisciplinary meeting using established clinical criteria [12–16] without knowledge of PET or cerebrospinal fluid (CSF) results or *APOE* carrier status. Clinical syndrome (sDx: subjective cognitive decline, (SCD), mild cognitive impairment (MCI) or dementia) and the suspected primary etiology (eDx: Alzheimer’s disease (AD), vascular, frontotemporal lobal degeneration (FTLD), Lewy body dementia (DLB), other neurodegenerative or neurological diseases [e.g., corticobasal degeneration], or non-neurodegenerative [e.g., psychiatric or epilepsy]) were determined during a multidisciplinary meeting. Next, amyloid-PET results were disclosed to the managing physician, and confirmation or change in diagnosis was captured. The mean interval between initial dementia evaluation and [^18^F]flutemetamol amyloid-PET scan was 71 ± 136 days. When amyloid-PET results were disclosed, the managing physician responsible for the initial diagnosis re-evaluated the most probable diagnosis. Between baseline dementia evaluation and disclosure of amyloid-PET results, no other diagnostic test results were disclosed to the neurologist [5]. For this work, syndrome and etiological diagnosis after amyloid-PET disclosure and subsequent follow-up were used. As such, syndromic diagnosis at the baseline time point of the current work could deviate from the initially intended mild dementia recruitment aim. Importantly, follow-up diagnosis was determined by the managing physician only.

### PET acquisition and visual assessment

All [^18^F]flutemetamol amyloid-PET scans were acquired on a Gemini TF-64 PET/CT scanner (Philips Medical Systems, Best, the Netherlands). Patients first underwent a low-dose CT for attention correction purposes, followed by a 20-min PET-acquisition 90–110 min post-injection (p.i.) of 191 ± 10 MBq [^18^F]flutemetamol (i.e., 4 frames of 5 min). Scans were checked for movement and frames were summed to obtain a static (20-min) image for visual assessment. Using the Vinci 2.56 software, scans were rated as either amyloid *positive* (i.e., unilateral uptake in at least one cortical region or in the striatum) or amyloid *negative* (i.e., primarily white matter uptake) by a local nuclear medicine physician trained according to the manufacturer’s guidelines (https://www.readvizamyl.com/), who was blinded to clinical information, except for brain MRI.

### Cognitive assessments and clinical follow-up

For the whole cohort (*N* = 200), information on survival was available with a mean follow-up time of 6.7 years (*SD* = 2.2, range = 1.1–9.3). In a subset of 108 patients, longitudinal cognitive scores and clinical etiological diagnosis (eDx) by the managing physician at least 1 year after amyloid-PET acquisition were available, with a mean follow-up time of 3.06 years (*SD* = 1.23, range = 1.00–7.02). Neuropsychological tests were acquired annually to measure functioning across cognitive domains. Global cognitive functioning was assessed with the Mini-Mental State Examination (MMSE) [17]. Two tests for memory were used; verbal memory (immediate recall) using the Dutch version of the Rey Auditory Verbal Learning Test (i.e., 15-word test total score) and episodic memory using the Visual Association Test (VAT) [18]. The 1-min animal category fluency test was used to assess Language [19], the Stroop third panel for Executive Functioning [20], and the trail-making-test A for Attention [21]. Stability in clinical diagnosis was determined based on an agreement between baseline and the latest available eDx.

### Post-mortem assessments

Neuropathological diagnosis was available for 4 patients and performed according to NIA-AA guidelines. The extent of Alzheimer’s disease pathology was summarized by the ‘ABC score’, which is a composite of three scores: A for amyloid-beta (Aβ) Thal phase, B for Braak stage of neurofibrillary tangles, and C for Consortium to Establish a Registry for Alzheimer Disease (CERAD) score of neuritic plaques [22]. The presence of cerebral amyloid angiopathy (CAA) and α-synuclein pathology according to Braak stages and TAR DNA binding Protein (TDP) 43 was also assessed. The different stainings were performed according to the Brain Net Europe (BNE) guidelines by Alafuzoff et al., (2008a [23], 2008b [24], and 2009 [25]).

### Statistical analyses

All statistical analyses were performed in R version 4.0.2 and significance was set at 2-sided *p* < 0.05.

Baseline demographics and stability in eDx were assessed with a t-test, chi-square test, or percentage change, when applicable. The difference in survival rate between amyloid-negative and amyloid-positive patients was determined with survival analysis and Cox regression model (R *survival* package), with the latter corrected for baseline age, sex, sDx, and eDx.

To assess whether amyloid status predicted cognitive functioning (scores on 6 neuropsychological tests defined above) over time, linear mixed models with random intercept and slope were fitted (R *lme4* package). The main predictors were Aβ_status_, time, and their interaction, while covariates were age at baseline, sex, level of education, sDx, and eDx.

As a sensitivity analysis, the Cox regression model and linear mixed models were additionally performed for only the dementia group. Normality was assessed using the proportional-hazards (PH) assumption (R *survival* package) and Shapiro–Wilk (SW) test (R *LmerTest* package).

## Results

At baseline, patients were on average 61.8 years of age (*SD* = 5.8), 91 (45.5%) were female, mean MMSE was 23.3 (*SD* = 3.5), and 128 (64.0%) were visually assessed as amyloid-positive. Overall, 184 (92.0%) were considered to have dementia and 16 (8.0%) did not fulfill a diagnosis of dementia. Across diagnostic groups, 124 (62.0%) patients received a primary etiological diagnosis of AD, 3 (1.5%) vascular, 30 (15.0%) FTLD, 11 (5.5%) DLB, 9 (4.5%) other neurodegenerative disease, and 23 (11.5%) non-neurodegenerative disease. The majority of amyloid-positive patients had a primary etiological diagnosis of AD (122/128, 95.3%), while the amyloid-negative group consisted mostly of non-AD etiologies, particularly FTLD (30/72, 40.2%). Demographics did not differ for the subset of 108 patients with available longitudinal cognition and clinical etiological diagnosis (Table 1).

**Table 1:** Demographics of the cohort

	***Complete dataset***			***Subset with longitudinal clinical and cognition data***		
	**Total** **(*N* = 200)**	**VR − ** **(*N* = 72)**	**VR + ** **(*N* = 128)**	**Total** **(*N* = 108)**	**VR − ** **(*N* = 35)**	**VR + ** **(*N* = 73)**
**Age**	61.75 ± 5.8	61.63 ± 5.5	61.82 ± 6.0	61.6 ± 5.9	62.3 ± 5.4	61.2 ± 6.1
**Sex (F)**	91 (45.5%)	23 (31.9%)	68 (53.1%)	48 (44.4%)	11 (31.4%)	37 (50.7%)
***APOE*-ε4 carriership**	111 (55.5%)	25 (34.7%)	86 (67.2%)	89 (60.2%)	13 (39.4%)	52 (76.5%)
**Clinical diagnosis (post-PET)**						
SCD	4 (2.0%)	4 (5.6%)	n/a	n/a	n/a	n/a
MCI	12 (6.0%)	11 (15.3%)	1 (0.8%)	8 (7.4%)	7 (20.0%)	1 (1.4%)
Dementia	184 (92.0%)	57 (79.2%)	127 (99.2%)	100 (92.6%)	28 (80.0%)	72 (98.6%)
**Etiological diagnosis (post-PET)**						
AD	124 (62.0%)	2 (2.8%)	122 (95.3%)	71 (65.7%)	1 (2.9%)	70 (95.9%)
Vascular	3 (1.5%)	3 (4.2%)	n/a	2 (1.9%)	2 (5.7%)	n/a
FTLD	30 (15.0%)	29 (40.3%)	1 (0.8%)	13 (12.0%)	13 (37.1%)	n/a
DLB	11 (5.5%)	7 (9.7%)	4 (3.1%)	7 (6.5%)	4 (11.4%)	3 (4.1%)
ND other	9 (4.5%)	8 (11.1%)	1 (0.8%)	4 (3.7%)	4 (11.4%)	n/a
Non-ND	23 (11.5%)	23 (31.9%)	n/a	11 (10.2%)	11 (31.4%)	n/a

*SCD* subjective cognitive decline, *MCI* mild cognitive impairment, *AD* Alzheimer’s disease, *FTLD* frontotemporal lobar degeneration, *DLB* Lewy body disease, *ND* neurodegeneration, *VR* visual read

### Survival rates

**Fig. 2:**
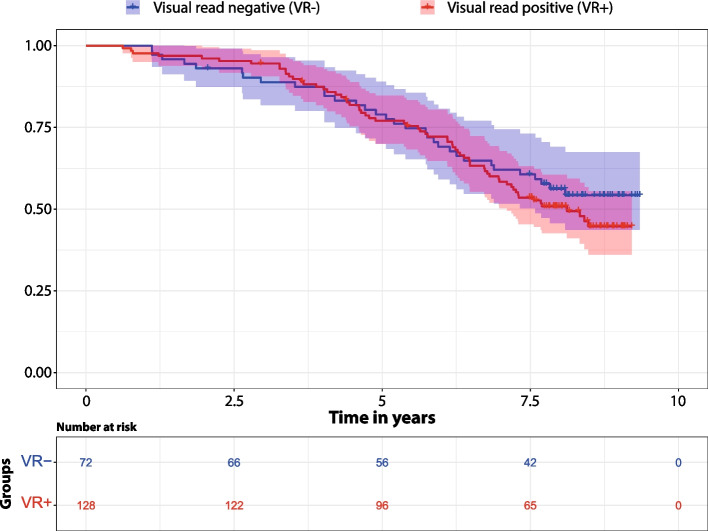
Kaplan–Meier survival curve Survival rate stratified by visual read (VR) status, showing no difference between visually negative and positive patients. The number of patients at each time point is also provided. Shaded represent the 95% confidence interval

The overall mortality rate was 48.5% in the whole cohort, with a mean follow-up time of 6.7 years. Mortality rate did not significantly differ between patients with AD and FTLD (49.2% vs 56.6%, *χ*^2^ = 0.28, *p* = 0.60), the two most prevalent groups. Mortality rates and average time to mortality did not differ between amyloid-negative and amyloid-positive patients (Aβ − : 44.5%, 4.5 ± 2.1 years vs Aβ + : 50.8%, 5.3 ± 2.0 years, *p* = 0.42 and *p* = 0.32, respectively, PH assumption: *p* = 0.37, Fig. 2). This was confirmed based on the Cox regression analysis, corrected for baseline age, sex, sDx, and eDx. Only sDx was a significant predictor of mortality, with patients with dementia showing a higher risk of death at follow-up compared to non-dementia patients (HR = 3.90, 95% CI:1.15–13.21, *p* = 0.03). Results were consistent when including only patients with dementia (VR − vs VR + : *p* = 0.71).

### Stability of diagnosis

**Fig. 3:**
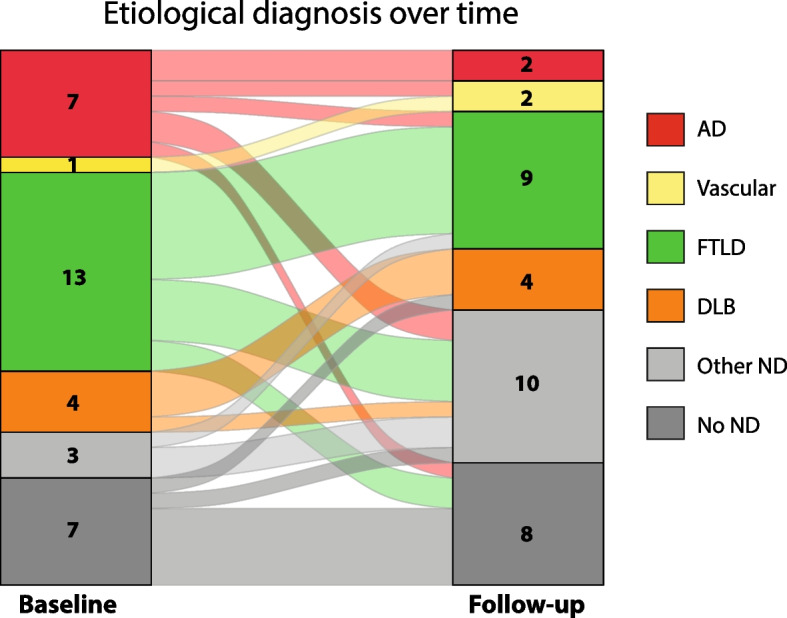
Flow diagram of changes in the etiological diagnosis of visually amyloid-negative cases AD, Alzheimer’s disease; FTLD, frontotemporal lobar degeneration; DLB, Lewy body disease; ND, neurodegeneration

In the subset with longitudinal cognition and clinical information available (*N* = 108, Table 1), etiological diagnosis at follow-up (last available) was consistent with baseline diagnosis (post amyloid-PET disclosure to physician) for 92 out of 108 patients (85.2%), with most changes observed in those cases assessed as amyloid-negative at baseline (Aβ −  = 14/35, 40% vs Aβ +  = 2/73, 2.7%, *χ*^2^ = 26.03, *p* < 0.001). Regarding the two amyloid-positive patients, one changed the diagnosis from DLB to AD and one from AD to “other ND,” in this case, corticobasal degeneration (CBD). Within the amyloid-negative group, 7/35 still had a primary etiological diagnosis of AD at baseline after disclosure of the amyloid-PET status. The etiological diagnosis changed at follow-up for 5 out of these 7 cases, with 2 cases receiving a “dementia other” diagnosis, 1 primary psychiatric disorder, 1 FTLD, and 1 primary vascular etiology (Fig. 3; red stream). Change in diagnosis at follow-up was also commonly observed in the VR-negative FTLD patient group, with about half of the patients (6/13) reclassified as “other ND” (mostly CBD) or primary psychiatric disorder (captured in “no neurodegeneration” category in Fig. 3; green stream).

### Cognitive functioning over time

**Fig. 4:**
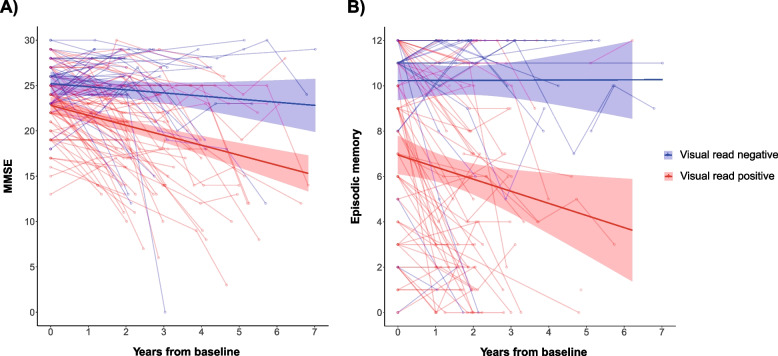
Longitudinal cognitive decline depends on visual read status Spaghetti plot illustrating the results of the linear mixed models. **A** Visually amyloid-positive subjects (red) show a steeper decline in global cognitive functioning as measured with the MMSE and **B** in episodic memory (visual association test) compared to visually amyloid-negative (blue) patients

In the same subset as above, amyloid-positivity was associated with a steeper decline over time across several neuropsychological tests and cognitive domains, after correction of key demographics, clinical stage, and etiological diagnosis. Amyloid-positivity was predictive of a steeper decline in global cognitive functioning as measured with the MMSE (*β* =  − 1.17, *p* = 0.004, Fig. 4A), episodic memory (VAT: *β* =  − 0.78, *p* = 0.003, Fig. 4B), fluency (*β* =  − 1.44, *p* < 0.001), and attention (TMT-A: *β* = 16.76, *p* = 0.03). In this sample of mostly patients with early-onset dementia, amyloid status was not predictive of a decline in memory recall (15-word test) and executive functioning (stroop3). Within the dementia population (*N* = 326), amyloid-positivity remained predictive of episodic memory (VAT: *β* =  − 0.70, *p* = 0.02) and fluency (*β* =  − 1.08,* p* = 0.02), while its effect on global cognition (MMSE: *β* =  − 0.86, *p* = 0.057) and attention (TMT-A: *β* = 15.82, *p* = 0.06) was reduced to trend level. The normality assumption was not met for these models (SW-test: *p* > 0.05).

### Post-mortem confirmation

**Fig. 5:**
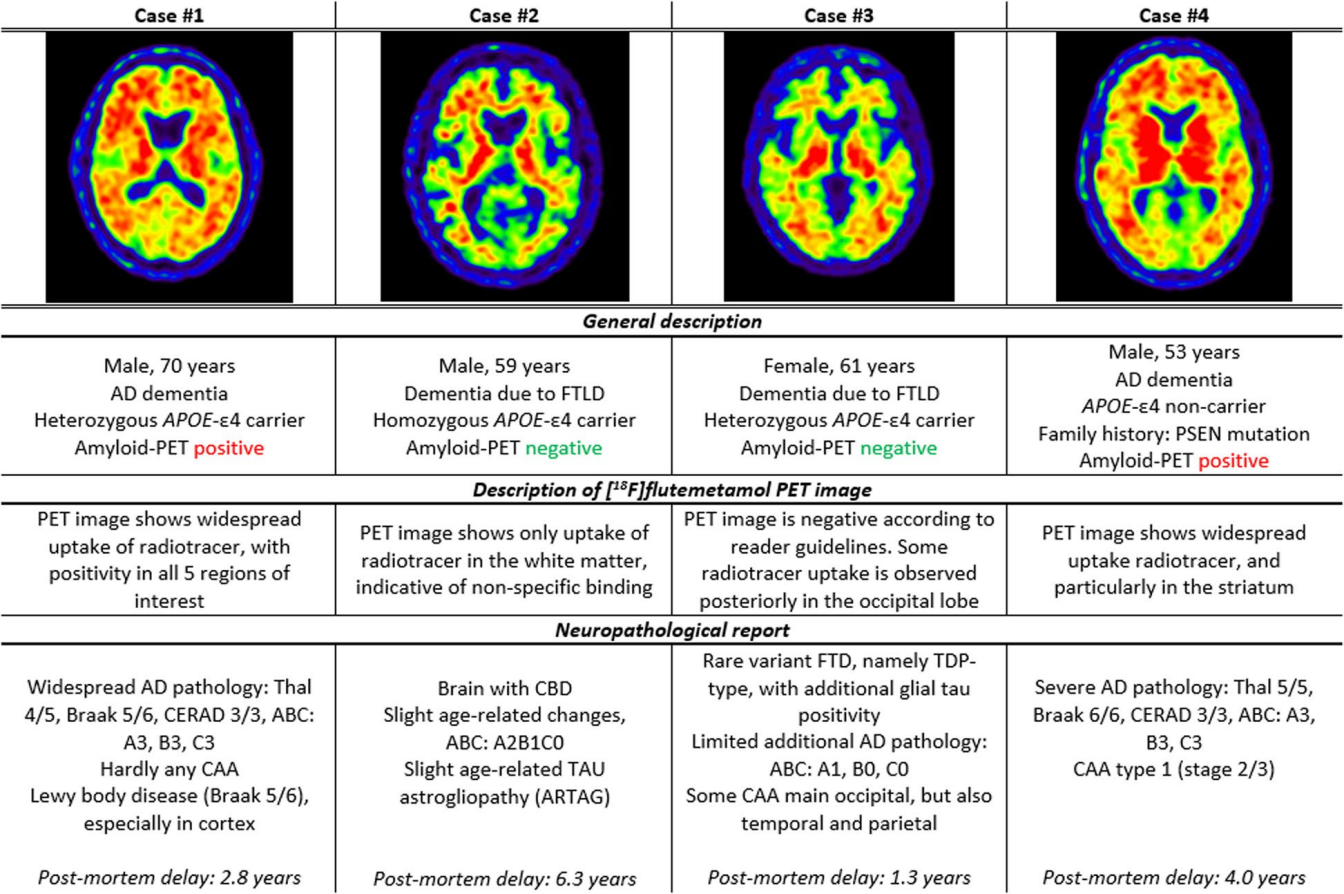
Post-mortem cases CAA, cerebral amyloid angiopathy; PSEN, presenilin; AD, Alzheimer’s disease; FTLD, frontotemporal lobar degeneration; CBD, corticobasal degeneration

Neuropathological data was available for 4 patients. Mean delay between amyloid-PET acquisition and date of death was 3.59 years (± 1.82, range = 1.2–6.3). Amyloid-PET assessment was in line with *post-mortem* confirmation in all cases. More specifically, two cases were visually assessed as amyloid-positive based on the PET scan and both showed widespread AD pathology at *post-mortem* examination. The other two cases were assessed as visually amyloid-negative based on the PET scan, with one case receiving a neuropathological diagnosis of a TDP-type variant of FTLD and displaying limited amyloid pathology only (A1) at *post-mortem* assessment, while the other received a neuropathological diagnosis of corticobasal degeneration (CBD) and described to have some age-related AD pathological changes (A2, B1), though this case had the longest interval between amyloid-PET imaging and autopsy (6.3 years). A detailed description of the cases is provided in Fig. 5.

## Discussion

In the current work, we investigated longitudinal clinical outcome measures in a population consisting primarily of early-onset dementia patients who underwent amyloid-PET imaging during their initial diagnostic work-up. We observed an overall mortality rate of 48.5% with a mean follow-up duration of 6.7 years, which did not differ between amyloid-negative and amyloid-positive patients based on visual assessment. Nonetheless, amyloid-positive patients did show a steeper decline in global cognitive functioning, episodic memory, fluency, and attention. Also, etiological diagnosis at follow-up was highly consistent with amyloid-PET status. In particular, we further demonstrate the excellent negative predictive value of amyloid-PET, as amyloid-negative patients did not receive an AD diagnosis at follow-up. Finally, visual assessment was in concordance with neuropathological scores.

While clinical- and patient-centered outcomes directly following amyloid-PET are quite abundantly available, longitudinal outcomes are scarce [8]. By design, the ultimate target outcomes of patient health and well-being, i.e., mortality, have been left largely unexplored. Previous work has demonstrated that the average life expectancy of patients diagnosed in their 60 s and early 70 s with AD dementia could be as long as 7 to 10 years, requiring an extensive follow-up period [26]. With our mean follow-up time of almost 7 and up to 9 years, we uniquely demonstrate the mortality rate in a symptomatic memory-clinic-based population suspected of early-onset dementia stratified by amyloid-PET status. The overall mortality rate in our study was 48.5%, which is highly comparable to a previous publication based on the whole Amsterdam Dementia Cohort (ADC) [27]. Mortality rate did not differ based on amyloid-PET visual read status and only syndrome diagnosis was a significant contributor to survival, with patients with dementia showing a higher risk of death at follow-up compared to non-demented patients. This latter finding is in line with a review illustrating that particularly disease severity was associated with increased risk of mortality [28] and the previous work based on the ADC cohort [27]. The lack of amyloid-status associated risk is in line with a previous observational study, which suggested that while predictor variables characteristic of AD increase the hazard of dementia, mortality rates are not highly dependent on the specific etiology once the dementia stage is reached [9]. Nevertheless, the composition of our early-onset dementia population could also explain the lack of findings, considering that our amyloid-negative group was overrepresented by FTLD cases. Previous work demonstrated that particularly this patient population shows a rapid decline and worse prognosis compared to AD and might obscure any effect of amyloid positivity [29]. It would therefore be of interest to investigate survival rates in patients with a primary non-AD etiological diagnosis not enriched for FTLD, with and without concomitant amyloid pathology. Indeed, previous work already illustrated shorter survival times for DLB patients with additional amyloid pathology [30] or hippocampal atrophy as a proxy of AD pathology [31]. This specific analysis was unfortunately not possible in the current study due to sample size of DLB cases, though all amyloid-positive DLB cases (*N* = 4) were deceased at follow-up (range 3.6–6.2 years), which was not the case for their amyloid-negative counterparts (*N* = 7, 100% survival rate over 8.0–9.3 years of follow-up). In line, we observed that amyloid-status was associated with longitudinal cognitive functioning, with a stronger decline in several cognitive domains for amyloid-positive patients.

Amyloid status was also associated with the stability of etiological diagnosis at follow-up. In line with previous work [32], we observed a minimal change in diagnosis for amyloid-positive patients over an average of 3 and up to 7 years. Instead, most changes were observed in patients with a negative amyloid-PET assessment and non-AD as the primary diagnosis, stressing the need for more disease-specific biomarkers. Importantly, the assessment of the amyloid-PET images was in concordance with the final neuropathological report available for 4 cases, though an interesting observation was made for case #3. This patient’s scan was assessed as negative; however, some radiotracer uptake can be appreciated in the posterior areas of the brain. The occipital lobe is not considered a region of interest in the [^18^F]flutemetamol reader guidelines, but uptake in this region has been associated with cerebral amyloid angiopathy (CAA) [33, 34], which was also observed at *post-mortem* evaluation for this particular patient. This suggests that regional patterns of amyloid uptake might further contribute to the diagnostic work-up and possibly provide information on comorbidities, which is commonly observed in AD patients [35].

Our results regarding clinician- and patient-centered outcomes could be combined with health-economics data to aid with country-wide economic forecasting associated with the overall dementia care cost burden. A recent meta-analysis estimated annual costs ranging from 8000 EUR (Eastern Europe) up to 70,000 EUR (UK) for patients with dementia across European countries and costs were considerably higher for institutionalized patients and for those with more severe disease [36]. In the Western European countries including the Netherlands where the analysis for this paper was performed the average costs were approximately 38,000 Euros. Most previous studies have focused on the costs and benefits immediately related to the diagnostic procedure over a relatively short period of time [8]. However, several projects, such as ABIDE [37], IDEAS [38], and AMYPAD [39] aim to provide further evidence on the cost-utility of amyloid-PET in the clinical routine. In fact, a recent publication of the ABIDE-PET study showed that total health-care costs after diagnosis were lower in the amyloid-PET group compared to the no-PET group, being the first study to provide evidence that a precise and timely diagnosis may contribute to better health outcomes [40].

The current work has some methodological considerations and limitations. First, the current work is based on a single-center study, limiting the generalizability of the results. However, the main outcome measure (i.e., clinical diagnosis) is as such highly standardized and enabled assessment over an extended period of time. Also, the cohort is heterogeneous in its composition regarding etiologies underlying dementia, reflecting real-world clinical routine. Secondly, the cohort consists of mainly early-onset dementia patients, limiting generalizability to late-onset populations. Thirdly, clinical outcome was restricted to mortality rate, as other clinical endpoints, such as change in patient management or hospice, were only sporadically collected over the extended follow-up period. Also, the mortality survival analysis did not take the possible presence of comorbidities into account. Regarding the linear mixed models, these did not meet the normality assumption. However, linear mixed models are particularly robust to handle non-normal data [41] and therefore implemented in the current work. Finally, follow-up etiological diagnosis was provided by the managing physician only, while baseline diagnosis was provided by an expert panel.

## Conclusion

In a symptomatic population consisting of mostly patients with early-onset dementia who underwent amyloid-PET imaging for their diagnostic work-up, we report unique longitudinal information on survival rates, cognitive decline, stability of etiological diagnosis, and *post-mortem* confirmation. Data provided in this work further illustrates the clinical validity of amyloid-PET imaging.

## Data Availability

Data can be made available upon reasonable request.
